# Cognitive Assessment in HTLV-1 Patients Followed Up at a Reference Center in Salvador, Brazil

**DOI:** 10.3390/v16101569

**Published:** 2024-10-05

**Authors:** Luísa Bordallo, Iris Montaño-Castellón, Liliane Lins-Kusterer, Carlos Brites

**Affiliations:** 1 School of Medicine, Federal University of Bahia, Salvador 40110-100, Brazil; luisa.bordallo@ufba.br; 2 Post-Graduation Program in Health Sciences, School of Medicine, Federal University of Bahia, Salvador 40110-060, Brazil; iris.med.umss@gmail.com; 3 Department of Preventive and Social Medicine, School of Medicine, Federal University of Bahia, Salvador 40110-100, Brazil; liliane.lins@ufba.br; 4 Department of Medicine and Diagnostic Support, School of Medicine, Federal University of Bahia, Salvador 40110-100, Brazil

**Keywords:** HTLV-1, cognitive dysfunction, depression

## Abstract

Introduction: Human T-cell lymphotropic virus type 1 (HTLV-1) is endemic to Brazil, and there is still no specific treatment for these patients. The literature shows that few studies have described the cognitive impairment associated with an HTLV-1 infection, with none of them examining the population of Salvador, where there are approximately forty thousand people infected with the virus. Objectives: To determine the prevalence of cognitive impairment among individuals with HTLV-1. In addition, investigate whether sociodemographic aspects, time since the diagnosis of infection, and the diagnosis of HTLV-Associated Myelopatia/Tropical Spastic Paraparesis (HAM/TSP) or depression are associated with cognitive impairment in this population. Methods: This was an observational, cross-sectional study that consisted of consecutively approaching 100 HTLV-1 patients during outpatient care at a referral center followed by the administration of three questionnaires— the Mini Mental State Examination (MMSE), the Montreal Cognitive Assessment (MoCA), and Beck’s Depression Inventory. Results: The prevalence of cognitive impairment found was 71% using the MMSE and 82% using the MoCA. There was a statistically significant association between the cognitive dysfunction and the variables of age and education according to the MoCA analysis but not the MMSE data. Diagnosis of HAM/TSP was correlated with cognitive impairment using the MMSE but not the MoCA. The prevalence of depression was 20%, and there was no association between cognitive impairment and depressive symptoms in these patients. Conclusions: The findings of this study demonstrate a correlation between cognitive dysfunction and HTVL-1 infection, with a more evident involvement of executive functions and memory. Larger studies are needed to clarify the association between cognitive dysfunction, age, education, and the diagnosis of HAM/TSP.

## 1. Introduction

Human T-cell lymphotropic virus type 1 (HTLV-1) is endemic to Brazil and affects an estimated 800,000 people [1]. Salvador, the capital of the Bahia state, has one of the highest prevalences of HTLV infection in Brazil, with an estimated 40,000 people living with HTLV [2]. Despite HTLV-1 being the first human retrovirus discovered and affecting people worldwide, it is considered a neglected disease and the knowledge on its pathogenesis is still limited. No treatment is currently available for an HTLV infection [1]. The highest rates of this sexually transmitted infection are found in marginalized populations, such as low-income and low-educated individuals, sex workers, and injecting drug users, in rural or indigenous populations and in less developed countries [3]. In addition, some areas in developed countries (like Japan) present a high prevalence of HTLV-1 infection, mostly due to vertical transmission by breastfeeding.

HTLV-1 causes several diseases, like HTLV-1-associated myelopathy/tropical spastic paresis (HAM/TSP), adult T-cell leukemia (ATL), arthritis, uveitis, dermatitis, myositis, and sicca syndrome, among others [4,5,6,7,8,9]. HTLV has a preferential tropism for CD4+ T lymphocytes, and the development of clinical manifestations seems to be related to a higher proviral load and to an exacerbated inflammatory cell response, but many points on the pathogenesis of this infection are still unclear [10,11,12].

HAM/TSP is considered the most severe neurological manifestation associated with HTLV-1 due to its progressive character and disabling symptoms, which include spastic paraplegia/paraparesis, sensory deficit, and sphincter or sexual dysfunction [6,12]. Some studies have suggested a correlation between HTLV-1 infection and alterations in the central nervous system, such as alterations in the cerebral white matter seen on magnetic resonance imaging as well as cognitive disorders like those identified in patients with multiple sclerosis or individuals infected with HIV [8,13,14,15,16].

Cognitive dysfunctions have been well described in people with HIV, another retrovirus, especially in the pre-treatment era, and its neurotropism for central and peripheral nervous system is well known [17,18]. However, there are few studies on the cognitive impairment associated with an HTLV infection [8,16], and none of them have involved patients with the characteristics of the population of Salvador, an endemic city for this virus.

Our work aimed to assess the prevalence of cognitive impairment and describe the main cognitive domains affected in HTLV-1 patients as well as to evaluate if there is any association between the sociodemographic aspects, elapsed time since the HTLV-1 diagnosis, the diagnosis of HAM/TSP or depression, and cognitive impairment.

## 2. Materials and Methods

### 2.1. Study Location

We enrolled patients attended at the Professor Edgard Santos University Hospital (C-HUPES), a referral center for HTLV-1 care in Salvador. The HTLV outpatient clinic and the Infectious Diseases Research Laboratory of the hospital provide care to patients living with HTLV, including diagnostic support and the treatment of complications. There are around 1000 HTLV patients under regular follow-up at C-HUPES.

### 2.2. Design & Sampling

This is an observational, cross-sectional study that evaluated cognitive disorders in HTLV-1 patients followed up at C-HUPES. The inclusion criteria were age 18 years and older with a confirmed (by Western blot or PCR) HTLV-1 infection. Patients with a diagnosis of any previous comorbidity causing cognitive impairment or presenting with auditory/visual impairment were excluded. Consecutive patients attending the HTLV outpatients’ clinics were invited to the study until the intended sample was completed. The investigators did not know the list of patients scheduled for medical evaluation during the data collection days.

### 2.3. Procedures and Instruments

The following three questionnaires were used for cognitive evaluation: The Mini Mental State Examination (MMSE) [19], globally employed as one of the main screening tests for cognitive decline, whose cut-off marks depend on the level of education [20]. The Montreal Cognitive Assessment (MoCA) [21], developed or the global assessment of cognition, is more sensitive to mild cognitive deficits. In this test, one point is added for individuals with less than 11 years of schooling, and scores between 24 and 26 are considered insufficient to determine the presence or absence of a cognitive deficit [22]. The maximum score for both tests is 30. Finally, the Beck’s Depression Inventory (BDI) [23], created to establish the presence and severity of depressive symptoms. A score of higher than 20 points is considered indicative of depression [24].

The overall score in each of the tests and the specific scores were considered for five cognitive domains in the MMSE (orientation, immediate memory, attention and calculation, recall, and language) and eight in the MoCA (visuospatial/executive, naming, attention and calculation, language, abstraction, delayed recall, and orientation). The cut-off scores for each test are described in Table 1.

Clinical and sociodemographic data were obtained through a review of the medical charts. The questionnaires were administered by a trained neurologist and a trained medical student.

### 2.4. Statistical Analysis

The descriptive analysis for continuous variables was performed with mean and standard deviation (according to the Shapiro–Wilk normality test) and categorical variables were expressed as frequencies and absolute numbers. Associations between categorical variables were assessed using the chi-squared or Fisher’s exact test, when appropriate. The continuous variable “age” was compared to the MMSE data using Student’s T test and to the MoCA data using Welch’s ANOVA. The non-parametric continuous variable “elapsed time since HTLV-1 diagnosis” was compared to the MMSE data using the Mann–Whitney U test and to the MoCA data using the Kruskal–Wallis test. *p*-values under 0.05 were considered statistically significant. The software used was Jamovi version 2.3 (available at: https://www.jamovi.org/download.html, last accessed on 17 July 2024).

### 2.5. Ethical Aspects

The study was conducted according to the Declaration of Helsinki, the Guidelines for Good Clinical Practice, Protection of Human Subjects, and Data Protection, and the national regulations.

An informed consent form was signed by all participants before entering the study. The study protocol was approved by the Human Research Ethics Committee of the Professor Edgard Santos University Hospital (Number 69605123.2.0000.0049).

## 3. Results

Among the 100 patients included in the study, there was a higher percentage of women (73%), most (53%) self-identified as mixed-race or black (42%), the mean age was 54.14 ± 12.7 years, and 55% reported less than 11 years of schooling. The mean elapsed time since the diagnosis of HTLV-1 was 11.05 ± 7.84 years, and 61% of the patients had a HAM/TSP diagnosis. The demographic profile of the sample according to sex, age, race, schooling, and elapsed time since HTLV-1 diagnosis are described in Table 2.

Cognitive dysfunction was detected in 71% of the patients by the MMSE, predominantly among the individuals diagnosed with HAM/TSP (*p* = 0.041). There was a marginally significant association between cognitive deficit and education (*p* = 0.071), and no significant association was found with the other variables analyzed, as can be seen in Table 3.

The MoCA test detected an 82% prevalence of cognitive deficit. In addition, 13% of the sample had an intermediate score, indicating that further investigations are required to determine the presence or absence of cognitive dysfunction. Also, a statistically significant association was detected between cognitive impairment, age (*p* = 0.034), and low education (*p* = 0.035), as described in Table 4.

The overall prevalence of depression was 20%, and no association was identified between cognitive dysfunction and depression in either the MMSE (*p* = 0.495) or the MoCA (*p* = 0.823).

## 4. Discussion

A high prevalence of cognitive deficit was detected among the people living with HTLV-1 in both the MMSE (71%) and MoCA (82%) questionnaires. The difference between the prevalences found by the tests can be attributed to the MoCA’s higher sensitivity to detect early stages of cognitive decline [25], which suggests that the value found through the MMSE application is underestimated. Given that cognitive decline is a significant predictor of functional dependence [25], these findings point to an important limitation on the quality of life, personal, academic and work performance, social role, and self-perception of these patients.

A significant association was identified between the presence of cognitive deficit, age, and low schooling in the MoCA analysis, which suggests that aging and low schooling may have influenced the results [26]. Accordingly, in the MMSE data, there was a suggestion of a residual effect of schooling as a predictor of cognitive dysfunction. The heterogeneity of basic education in Brazil indicates that individuals reporting the same time of schooling may have distinct levels of learning and performance [27]. This may interfere with the adequacy of the cut-off scores, especially in the MoCA, which are not yet well validated for individuals with low schooling or in populations with the characteristics of the sample used, i.e., Brazilians, mostly mixed race/black, with HTLV-1.

The results of the MMSE application suggest that the diagnosis of HAM/TSP is predictive of cognitive dysfunction, although there is a divergence between the data found through the MoCA and MMSE tools. This association between HAM/TSP and cognitive dysfunction deserves further investigations on the pathophysiology of viral involvement of the central nervous system, which is still poorly understood. No statistically significant associations were shown between cognitive dysfunction and gender, color/race, and elapsed time since the diagnosis of HTLV-1 infection.

Some limitations of the present study include the absence of a control group, its cross-sectional design, and small sample size. In addition, it was not a random sample. However, the fact that the study was conducted in a referral service provides greater sample homogeneity, and the detected prevalence of cognitive disturbances is relevant enough to justify the hypothesis that HTLV-1 infection is independently associated with cognitive impairment. The inclusion of consecutive patients also mitigates the lack of a randomized sample, as the investigator did not know the list of patients assigned to a specific day.

There was also evidence of a prevalence of depression that is much higher than that of the general population, which is estimated to be 4–5% in Brazil [28], and no association was found between cognitive impairment and depression in these patients. This scenario could be attributed to the stigma associated with the diagnosis of this sexually transmitted disease, the absence of specific treatment, or the functional limitation presented by symptomatic patients [28].

Considering that the cognitive performance of HIV patients has improved in the post-antiretroviral therapy era [18], the results of this study emphasize the demand for studies that seek specific therapeutic interventions for the population of people with HTLV-1, in addition to the importance of early diagnosis and primary prevention of this infection.

## 5. Conclusions

The findings of this study reinforce the hypothesis that there is a correlation between cognitive dysfunction and HTVL-1 infection and show more evident impairment of the patients’ executive functions and memory. The results indicate the need of multicenter studies to achieve a more robust and representative sample and to confirm the existence of an association between cognitive dysfunction and age, education, and diagnosis of HAM/TSP.

## Figures and Tables

**Table 1:** Cut-off scores of the questionnaires used in the study.

MMSE [20]	Illiterate	<20
	1–4 years of education	<25
	5–8 years of education	<26
	9–11 years of education	<28
	>11 years of education	<29
MoCA [22]	Absent	≥27
	Indeterminate	24–26
	Present	≤23
BDI [24]	Absent	≤20
	Present	>20

Abbreviations: Mini Mental State Examination (MMSE); Montreal Cognitive Assessment (MoCA); Beck’s Depression Inventory (BDI).

**Table 2:** Demographic characteristics of study’s population.

Sex	Female	73
	Male	27
Age, mean ± SD		54.14 years ± 12.7
Race	Mixed-race	53
	Black	42
	White	5
Education	Illiterate	4
	1–4 years	15
	5–8 years	24
	9–11 years	12
	>11 years	45
Elapsed time since HTLV-1 diagnosis, mean ± SD		11.05 years ± 7.84
HAM/TSP	Absent	39
	Present	61

Percentage values correspond to the absolute number, since *n* = 100; SD = standard deviation; Human T-cell lymphotropic virus type 1 (HTLV-1); HTLV associated myelopathy (HAM/TSP).

**Table 3:** Cognitive dysfunction using MMSE.

	Absent	Present	Total	*p*-Value
Age, mean (SD)	53.1 (±12.8)	54.6 (±12.8)		0.303
Sex				
Female	20	53	73	0.623
Male	9	18	27	
Race				
Mixed-race	14	39	53	0.307
Black	12	30	42	
White	3	2	5	
Education				
Illiterate	3	1	4	0.071
1–4 years	5	10	15	
5–8 years	10	14	24	
9–11 years	2	10	12	
>11 years	9	36	45	
Elapsed time since HTLV-1 diagnosis, mean (SD)	10.9 (±7.59)	11.1 (±8.00)		0.453
HAM/TSP				
Absent	7	32	39	0.041
Present	22	39	61	
Depression				
Absent	24	58	82	0.495
Present	5	13	18	
Total	29	71	100	

Percentage values correspond to the absolute number, since *n* = 100; SD = standard deviation. Human T-cell lymphotropic virus type 1 (HTLV-1); HTLV associated myelopathy (HAM/TSP).

**Table 4:** Cognitive dysfunction using MoCA.

	Absent	Indeterminate	Present	Total	*p*-Value
Age, mean (SD)	44.4 (±16.2)	44.8 (±12.9)	56.2 (±11.7)		0.034
Sex					
Female	3	10	60	73	0.806
Male	2	3	22	27	
Race					
Mixed-race	3	10	40	53	0.150
Black	1	3	38	42	
White	1	0	4	5	
Education					
Illiterate	0	0	4	4	0.035
1–4 years	0	1	14	15	
5–8 years	2	0	22	24	
9–11 years	1	0	11	12	
>11 years	2	12	31	45	
Elapsed time since HTLV-1 diagnosis, mean (SD)	13.4 (±9.40)	12.2 (±9.28)	10.7 (±7.57)		0.842
HAM/TSP					
Absent	2	5	32		1.000
Present	3	8	50		
Depression					
Absent	5	12	65	82	0.483
Present	0	1	17	18	
Total	5	13	82	100	

Percentage values correspond to the absolute number, since *n* = 100; SD = standard deviation. Human T-cell lymphotropic virus type 1 (HTLV-1); HTLV associated myelopathy (HAM/TSP).

## Data Availability

The raw data supporting the conclusions of this article will be made available by the authors on request.
